# Exploring the Phytochemical Diversity and Anti-Plasmodial Potential of *Artemisia annua* and *Artemisia afra* from Different Geographical Locations in Cameroon

**DOI:** 10.3390/molecules30030596

**Published:** 2025-01-28

**Authors:** Lahngong M. Shinyuy, Gisèle E. Loe, Olivia Jansen, Allison Ledoux, Benjamin Palmaerts, Lúcia Mamede, Naima Boussif, Olivier Bonnet, Bertin S. Enone, Sandra F. Noukimi, Abenwie S. Nchang, Kristiaan Demeyer, Annie Robert, Stephen M. Ghogomu, Jacob Souopgui, Eric Hallot, Michel Frederich

**Affiliations:** 1 Pharmacognosy Laboratory, Center of Interdisciplinary Research on Medicine (CIRM), University of Liège, Avenue Hippocrate 15, 4000 Liège, Belgiumolivier.bonnet@uliege.be (O.B.); 2 Laboratory of Pharmacochemical and Natural Pharmaceutical Substances, Doctoral Training Unit in Health Sciences, Faculty of Medicine and Pharmaceutical Sciences, University of Douala, Douala P.O. Box 2701, Cameroon; 3 Molecular and Cell Biology Laboratory (MCBL), Department of Biochemistry and Molecular Biology, Faculty of Science, University of Buea, Buea P.O. Box 063, Cameroon; 4 Remote Sensing and Geodata Unit, Institut Scientifique de Service Public (ISSeP), 4000 Liège, Belgium; 5 Laboratory of In Vitro Toxicology and Dermato-Cosmetology (IVTD), Department of Analytical, Applied Chemometrics and Molecular Modeling (FABI), Faculty of Medicine and Pharmacy, Vrije Universiteit of Brussel, 1050 Ixelles, Belgium; 6 Embryology and Biotechnology Laboratory, Université Libre de Bruxelles, 1050 Bruxelles, Belgium; 7 Department of Epidemiology and Biostatistics (EPID), Institute de Recherche Expérimentale et Clinique (IREC), Public Health School, Université Catholique de Louvain (UCLouvain), 1200 Brussels, Belgium

**Keywords:** artemisinin, malaria, *Artemisia annua*, Artemisia afra, *Plasmodium*, metabolite

## Abstract

In Cameroon, like in other African countries, infusions of *Artemisia afra* and *Artemisia annua* are widely used for the management of health-related problems, including malaria. The secondary metabolite contents of medicinal plants vary between different geographical regions and seasons, directly influencing their effectiveness in treating ailments. This study explores the phytochemical diversity and anti-plasmodial potential of *A. annua* and *A. afra* from distinct geographical locations within Cameroon, aiming to define the optimal chemical composition in terms of anti-plasmodial activity. Extracts were prepared from plants collected from diverse regions in Cameroon during both the rainy and dry seasons, and their metabolic contents were analyzed using Thin-Layer Chromatography (TLC), High Performance Liquid Chromatography (HPLC), and Gas Chromatography (GC). Their anti-plasmodial potential was assessed on a chloroquine-sensitive 3D7 *Plasmodium falciparum* strain. Additionally, the environmental parameters of the collecting sites were retrieved from multispectral satellite imagery. The activity profiles of the samples were associated with their environment, with distinct phytochemical compositions observed for each sample based on its geographical origin and season. Traces of artemisinin were detected in some of the *A. afra* samples, but it was present in the *A. annua* samples at a significantly higher concentration, especially in the rainy season samples (highest concentration in the Adamawa region, at 8.9% m/m artemisinin in the dry extract). Both plants are active at different levels, with *A. annua* more active due to the presence of artemisinin and *A. afra* probably active due to the presence of polyphenols. Both season and geographical location influence both plants’ metabolic contents and hence their antimalaria activity. These findings suggest that the selection of a suitable *Artemisia* sample for use as a potential antimalarial treatment should take into consideration its geographical origin and the period of collection.

## 1. Introduction

Malaria is a disease of public health importance caused by parasites of the *Plasmodium* family and transmitted by female Anopheles mosquitoes. In most countries where malaria is endemic, this disease disproportionately affects children under the age of five and pregnant women. Globally, over 250 million malaria cases and 608,000 deaths were reported in 2022 in 85 malaria-endemic countries, with an estimated additional 13.4 million increased cases between 2019 and 2021 [1]. Sub-Saharan African countries are the hardest-hit zones, with approximately 95% of the world’s malaria burden and 96% of the death rate, with over 80% of these being children under the age of five and pregnant women [1]. According to WHO data, 70% of all malaria cases are concentrated in 11 countries, with Cameroon accounting for over 2.7 million cases [1]. This can be related to increasing antimalarial drug resistance to all of the chemotherapeutic measures recommended at present, including “Artemisinin-based Combination Therapy” (ACT) [1]. The situation is further aggravated not only by Histidine-Rich Protein-2 gene (HRP2 gene) deletion [2], which makes a diagnosis problematic, but also by the widespread resistance of mosquito to insecticides, as well as the rapid spread of invasive vector species (*Anopheles stephensi*) from the Horn of Africa. The fight against this life-threatening disease is one of the goals of the World Health Organization’s (WHO) Global Technical Strategy for malaria to reduce transmission and achieve a world free of malaria by 2030. Natural products have been investigated as potential medications for diseases including malaria [3] and are used by 80% of the global population in primary health care [4]. *Artemisia afra* Jacq. ex Willd and *Artemisia annua* L are annual herbs belonging to the Asteraceae family that have a long history of use against fevers related to malaria [5,6]. Due to their complex mixtures of different phytochemicals, both plant species have proven to be effective in the clinical management of diseases as traditional remedies. Several studies have been reported indicating the effectiveness of both plants against malaria. For instance, in vitro studies, including clinical investigations, have shown that *A. afra,* despite being devoid of artemisinin or having a negligible artemisinin content [7], can exhibit good anti-plasmodial properties against erythrocytic forms (IC_50_ between 5 μg/mL and 15 μg/mL) [8], hepatic forms, and gametocidal stages of the malaria parasite [7,9,10,11,12,13]. The pharmacological properties of *A. afra* and *A. annua* were well documented in reviews by du Toit and Van de Kooy [11] and Soni et al. [13], respectively.

**Figure 1:**
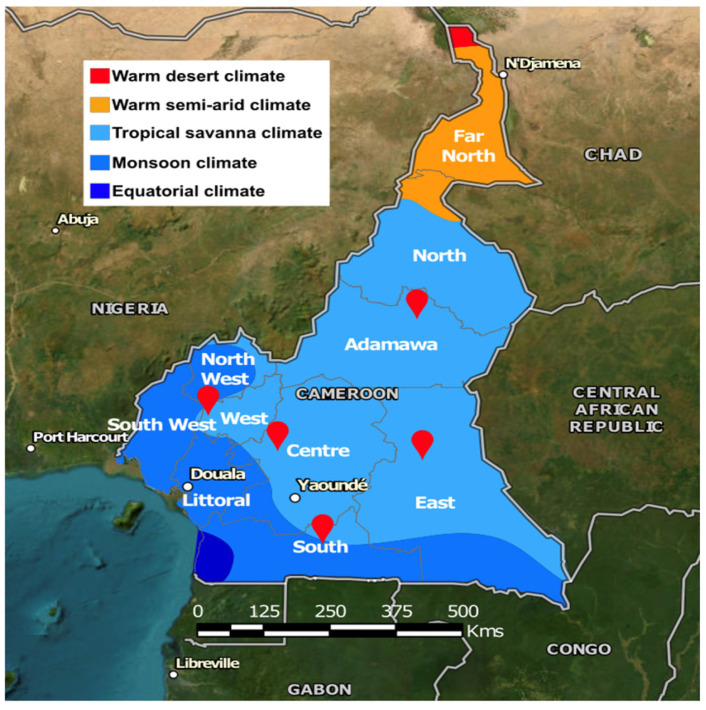
Location of the five collection sites. The map displays the administrative regions of Cameroon, and the colors indicate the climate type [17].

The isolation of artemisinin from *A. annua* [14], a potent antimalarial compound, has made the plant a subject of intensive phytochemical investigation in recent decades. Interestingly, phytochemical investigation has confirmed the isolation of almost 700 secondary metabolites from *A. annua* and almost 500 from *A. afra* [15]. The qualitative and quantitative contents of plant secondary metabolites are greatly influenced by temperature, humidity, soil type, soil micro-organisms, soil pH, and altitude [16]. This has a direct influence on a medicinal plant’s ability to effectively treat a well-diagnosed disease. In Cameroon, like in other African countries, *A. annua* and *A. afra* are cultivated in different geographical regions, formulated as herbal tea, and sold throughout several regions for the management of health-related problems, including malaria, but without pharmacological quality control. Additionally, given the close morphological similarity between the two *Artemisia* species, it is difficult to distinguish between the two medicinal plants physically (Supplementary Figure S1), and hence, discerning the authenticity of herbal tea is problematic. There is an absolute need to compare the metabolic contents and profiling of these plants obtained from different regions and during different cultivation periods and to develop a pharmaceutical monograph for plant authenticity and for quality control measures. This study explores the phytochemical diversity and anti-plasmodial potential of *A. annua* and *A. afra* sourced from distinct geographical locations in Cameroon. The chemical composition of these *Artemisia* species was investigated, aiming to discern potential variations in the phytochemical profiles attributable to geographic factors and correlated with observed activity, for plant authenticity, the optimal chemical composition, and quality control of these herbal products. To achieve this, both plant species were harvested in different seasons from different locations in Cameroon (Figure 1) and analyzed using TLC, HPLC, and GC. The environmental parameters of the collecting sites were retrieved from multispectral satellite imagery to monitor the health status of the vegetation where these plants were collected. Additionally, their anti-plasmodial activities were assessed, and a correlation was made between the observed activity and their metabolic fingerprints. The results of these findings show that the activity profiles of *Artemisia* plants are associated with their environment, with distinct phytochemical compositions observed for each plant sample based on its geographical origin and season of collection. Higher Normalized Different Vegetation Index (NDVI) values were observed during the rainy season regardless of the location of collection, indicating healthier and denser vegetation, which correlated with a higher artemisinin content (trend) and was associated with stronger antimalaria activity in *A. annua.*

## 2. Results

### 2.1. Comparison of TLC Fingerprints of Samples from Different Regions and Seasons

Thin-Layer Chromatography (TLC) fingerprints show inter-species and intra-species variation in the chemical patterns of metabolites. For example, we observed the presence of a pink-colored compound in all of the *A. afra* samples with a retention factor (RF) of 0.78, while this was absent in all of the *A. annua* extracts (Supplementary Figure S2a). The chemical patterns from TLC also indicate the absence of artemisinin in all of the *A. afra* extracts but its presence in the *A. annua* samples (RF = 0.51) (Supplementary Figure S2a). Additionally, the TLC fingerprints of the *A. afra* samples from different locations show intra-species variation in the chemical patterns, indicating the influence of harvest location on the chemical profile. For example, the *A. afra* extracts from the Center, South, and Adamawa regions show the same chemical pattern of terpenes, while the extracts from the West and East are characterized by a blue spot with different RF values and which is absent in the other samples (Supplementary Figure S2b).

The polyphenol fingerprints of both plants also show a distinct chemical pattern. For instance, luteolin and rutin are present in all *A. afra* samples but absent in the *A. annua* samples. Additionally, a region with black bands is detected in all *A. annua* samples though this is absent in *A. afra*. Samples of *A. afra* obtained from different regions show the same profile in terms of polyphenol composition (Supplementary Figure S2c). While seasonal variation was observed in the profile of terpene composition, there was no clear effect of season on polyphenol composition (Supplementary Figure S2d).

### 2.2. Influence of Season and Growing Location on the Natural Abundance of Major Polyphenols

**Figure 2:**
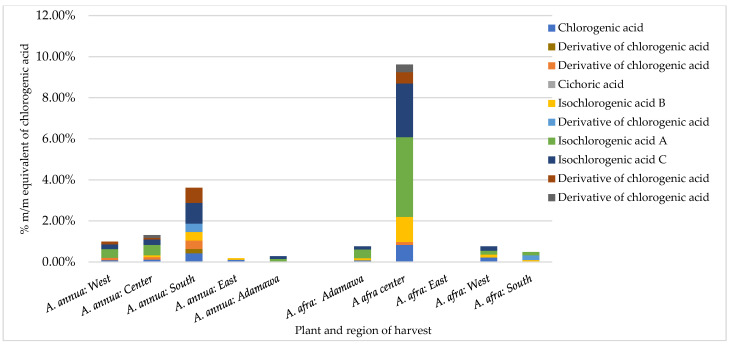
Contents of chlorogenic acid and its derivatives in *Artemisia* samples obtained in the rainy season expressed as % m/m equivalents of chlorogenic acid vary between different regions for both plants and inter-plant.

An HPLC analysis of polyphenols was performed on both series of samples harvested in the rainy and dry seasons. A reference calibration curve consisting of chlorogenic acid (0.1, 0.05, 0.025, and 0.0125 mg/mL in methanol) was made, and the polyphenol contents of 20 methanol crude extracts were estimated and expressed as % m/m equivalents of chlorogenic acid. Several polyphenols were identified in both samples through a comparison of the UV spectrum and retention time (Rt) of the analyte with those of standard reference compounds. These included rutin, chlorogenic acid, luteolin, astragalin, and iso-chlorogenic acid A, B, and C. In addition, coumarins (scopoline and scopoletin) were detected. The results obtained show inter- and intra-species variation in the concentration of chlorogenic acid (CA) and its derivatives (Figure 2). For the *A. afra* samples, the highest concentration of CA and its derivatives was found in the rainy season samples from the Center region (9.61% m/m chlorogenic acid equivalents), with iso-chlorogenic acid A being the major metabolite (3.89% m/m chlorogenic acid equivalents). In the sample from the East region, no detectable amount of CA nor its derivatives was retrieved. Within the *A. annua* samples, the concentration of CA and its derivatives ranged from 0.18 to 3.61% m/m CA equivalents in the samples from the East and South regions, respectively, indicating the effect of location on the CA contents. Additionally, while iso-chlorogenic acid A, B, and C were present in all samples, cichoric acid was only found in both species obtained from the Center region in the rainy season.

**Figure 3:**
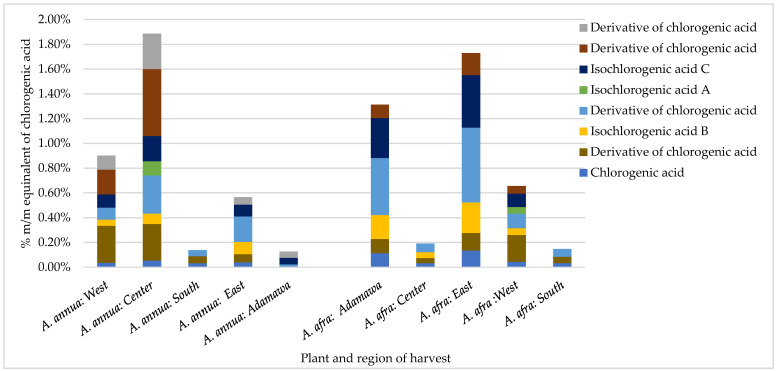
Effect of geographical location on the contents of chlorogenic acid and its derivatives in *Artemisia* samples obtained in the dry season expressed as % m/m equivalents of chlorogenic acid.

In addition to geographical variations, the results also show seasonal variations in the concentrations of CA and its derivatives. For example, while the *A. afra* samples obtained in the rainy season from the East region had no detectable amount of CA or its derivatives, the dry season samples presented high concentrations (1.73% m/m CA equivalents), with iso-chlorogenic acid B being one of the major phenolic acids (Figure 3). Except for *A. afra* from the Center region, generally, higher contents of CA and its derivatives were retrieved in the dry season samples compared to the rainy season samples. No detectable contents of cichoric acid were found in the dry season samples from both species. As for the *A. annua* samples, generally, higher contents of CA and its derivatives were also observed in the dry season, except from in the samples from the South region.

### 2.3. Influence of Season and Growing Location on the Natural Abundance of Major Semi-Volatile Components

**Figure 4:**
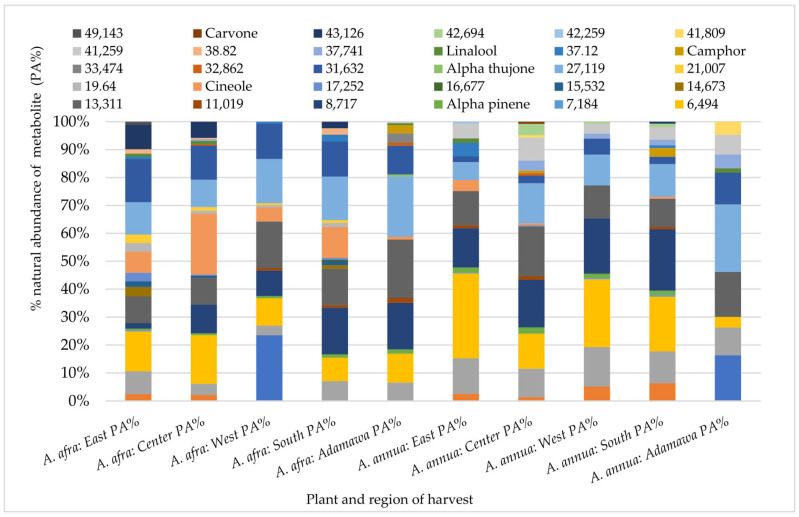
Influence of geographical location on the GC fingerprint and natural abundances of semi-volatile components in acetone extracts from both plants obtained from different regions in the rainy seasons. Each color indicates a metabolite, and the length gives an estimate of its relative occurrence. The numbers represent the corresponding retention times (RTs); PA% = peak area percent.

A Gas Chromatography (GC) analysis of semi-volatile components was performed using the acetone extracts of both plants obtained from different locations and seasons. Again, both inter and intra-species variations in the chemical pattern could be observed. Hence, indicating the effect of geographical location on the composition and natural abundances of the semi-volatile components of both species. While α-thujone was only identified in the *A. annua* samples from the Adamawa region, camphor, α-pinene and linalool were some of the metabolites identified in all rainy season samples with varying relative composition (Figure 4).

**Figure 5:**
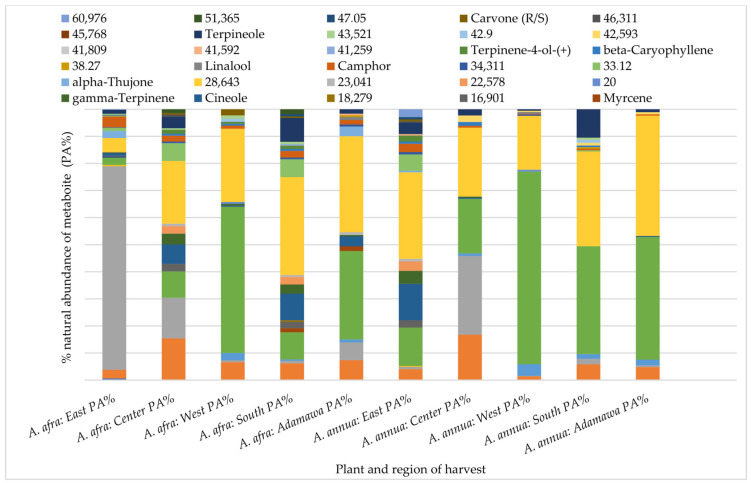
Variation in the GC fingerprint and natural abundances of semi-volatile components in acetone extracts from both plants obtained from different regions in the dry season. Each color indicates a metabolite, and the length gives an estimate of its relative occurrence. The numbers represent the corresponding retention times (RTs); PA% = peak area percent.

As for the samples obtained in the dry season, in addition to the locational variation, the results also show seasonal fluctuations in their composition. Carene-3-(+), terpineol, cineol, β-pinene, and camphor were some of the identified metabolites common to all of the dry season samples from both species but, again, with varying relative compositions (Figure 5).

### 2.4. Influence of Season and Growing Location on Artemisinin Content

**Figure 6:**
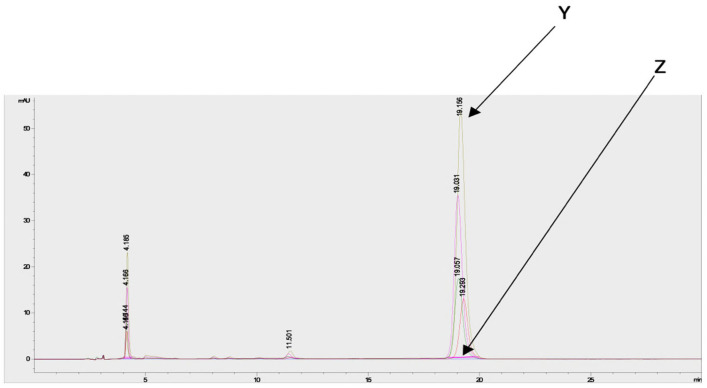
Chromatograms of artemisinin (standard) and derivatized and underivatized *A. annua* samples following HPLC analysis shows the presence of artemisinin in the *A. annua* samples. Y = chromatogram of derivatized samples and artemisinin, and Z = chromatogram of underivatized samples.

Estimation of the artemisinin contents in the acetone and methanol crude extracts of both species from different regions and seasons indicated a higher concentration in the acetone extracts compared to the methanol extracts (Supplementary Figure S3). Chromatograms of artemisinin (as the standard) and the derivatized and underivatized samples following the HPLC analysis are shown in Figure 6.

**Figure 7:**
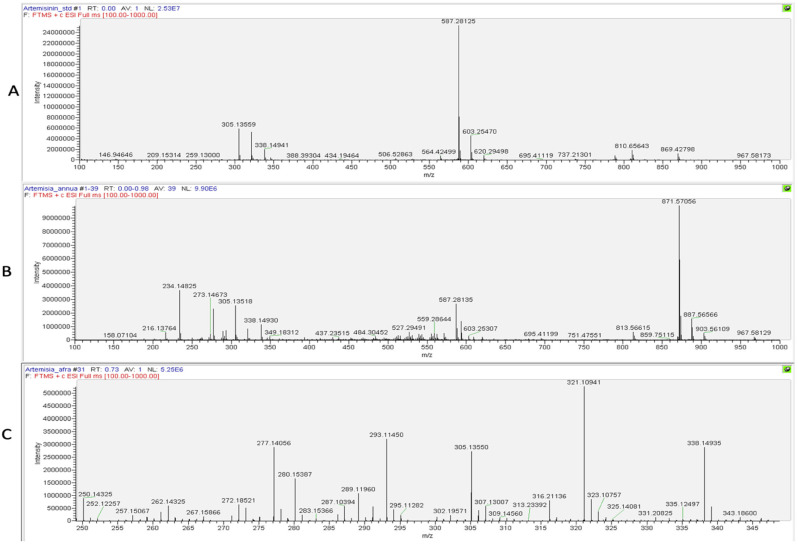
DI–HRMS spectra of (**A**) artemisinin, (**B**) *A. annua*, and (**C**) *A. afra* to visualize artemisinin [M+Na]+ contents.

The presence of artemisinin was further confirmed using a Direct Infusion High-Resolution Mass Spectrometry (DI–HRMS) analysis of the dry season samples of *A. annua* and *A. afra*, as derivatization can result in the production of artefacts. The spectrum for the artemisinin reference (Figure 7A) indicated the specific ions m/z 305.13550 ([M+Na]^+^) and m/z 587.28107 ([2M+Na]^+^), which were detected in the spectra of both the *A. annua* and *A. afra* samples using DI–HRMS (Figure 7B and Figure 7C, respectively).

**Figure 8:**
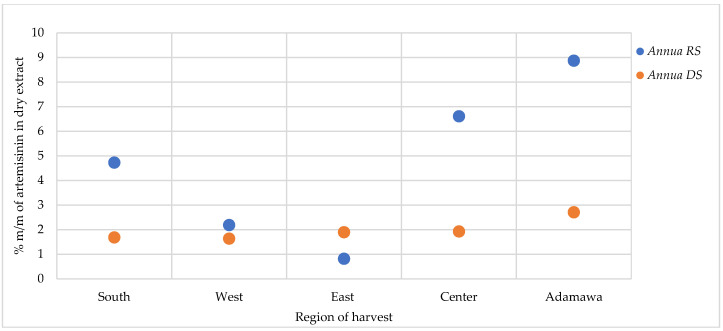
Effect of seasonal and geographical variation on artemisinin contents in *A. annua*. RS = rainy season samples, DS = dry season samples.

For the *A. afra* samples, only traces of artemisinin were found in some locations, while in other regions, no detectable amount could be found. As for *A. annua*, varying artemisinin contents were obtained as influenced by geographical and seasonal origin, with generally the highest contents detected in the rainy season samples (Figure 8). The *A. annua* samples from the Adamawa region showed the highest artemisinin content (8.88 and 2.71% for the rainy season and dry season samples, respectively), while the lowest contents were found in the samples from the East region.

### 2.5. Seasonal and Geographical Variation in the Total Polyphenol Contents

**Figure 9:**
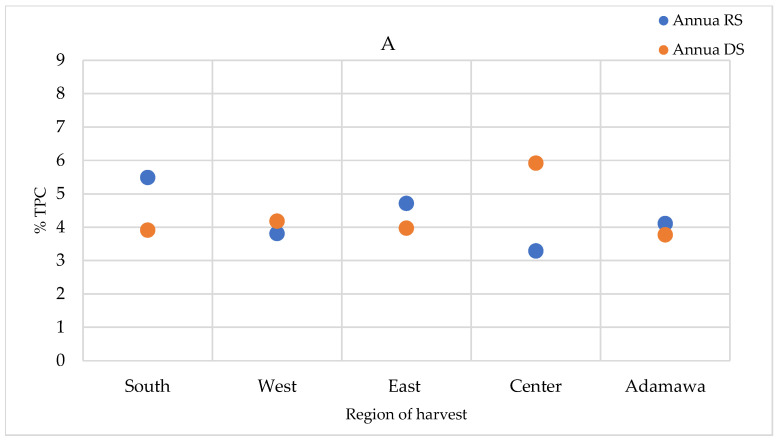

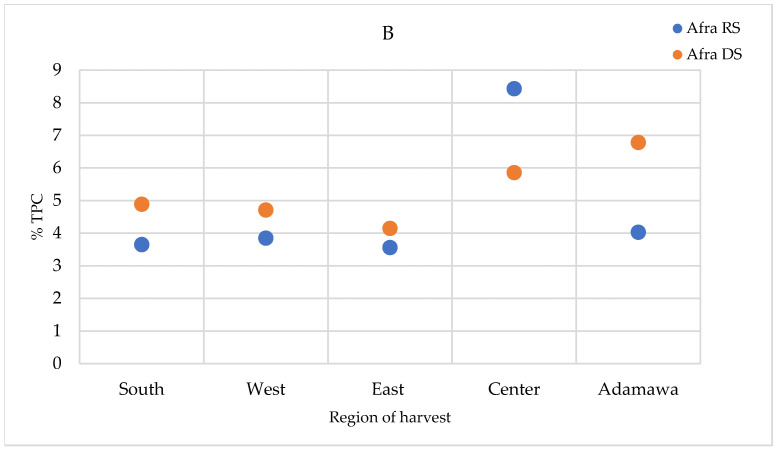
TPCs expressed as % m/m polyphenol in dry extract in methanol extracts of (**A**) *A. annua* and (**B**) *A. afra* obtained in the rainy and dry seasons. RS = rainy season samples; DS = dry season samples.

The total polyphenol contents (TPCs) were quantified in the extracts prepared using the infusion method (the traditional preparation), as well as the extracts obtained using the method described in the European Pharmacopoeia (heating and reflux) and in the methanol crude extracts. The TPCs were higher in the methanol crude extracts, followed by those obtained using the heating and reflux method, while infusion showed the lowest contents (Supplementary Figure S4). For the *A. annua* samples, only a relevant geographical fluctuation was ascertained (Figure 9A). However, for the *A. afra* samples, a seasonal effect was also observed, with the highest concentrations measured in the dry season samples, except for in the samples obtained from the Center region. The TPCs of *A. afra* ranged between 3.43 (in rainy season samples from the East region) and 8.43% m/m dry extract (in rainy season samples from the Center region) (Figure 9B). The values found within the *A. annua*-based extracts ranged between 3.29 and 5.92% m/m dry extract in the samples obtained from the Center region in the rainy and dry seasons, respectively (Figure 9A).

### 2.6. Anti-Plasmodial Activity of the Extracts and the Correlation with Chemical Patterns

**Figure 10:**
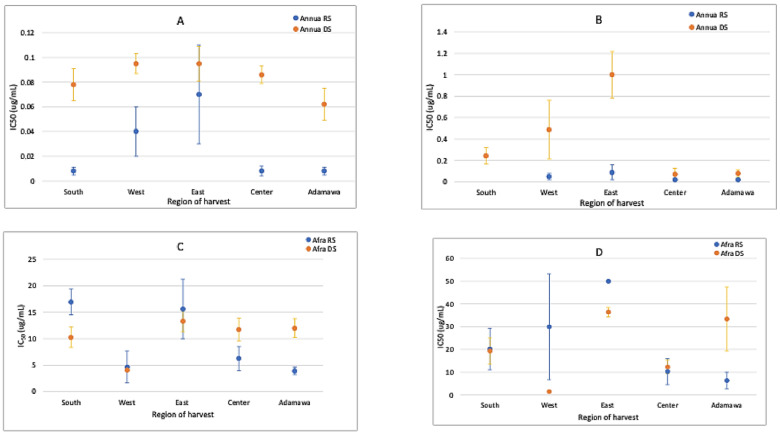
IC_50_ values (μg/mL) for (**A**) acetone extracts of *A. annua*, (**B**) methanol extracts of *A. annua*, (**C**) acetone extracts of *A. afra*, and (**D**) methanol extracts of *A. afra* from different regions and seasons show that both plants are active, with *A. annua* being more active (about a thousand-fold) than *A. afra*. The activity variates between the different seasons and geographical location. RS = rainy season; DS = dry season.

The anti-plasmodial activity of both plants was correlated with their metabolic fingerprints. Generally, the acetone extracts of both plants were more active than the methanol extracts (Figure 10). Both species were active against *Plasmodium* parasites. However, the *A. annua* extracts were more active than the *A. afra* extracts, with the activities fluctuating between samples from different regions and seasons. The rainy season samples of the *A. annua* extracts were more active than the dry season samples. This activity was artemisinin-dependent and related to the higher contents observed in the rainy season samples. The IC_50_ of artemisinin (standard drug) was 4.59 ± 1.15 ng/mL.

### 2.7. Correlation Between Environmental Conditions and Anti-Plasmodial Properties

**Figure 11:**
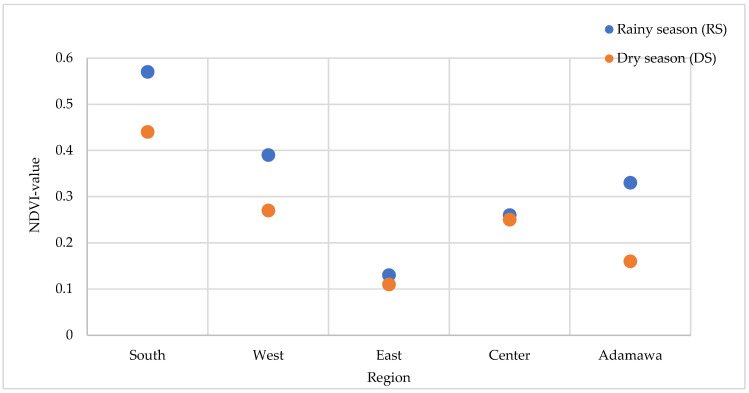
Variation in the NDVI values at the five collection sites and seasons.

Anti-plasmodial activity was compared for both seasons with the health status of the environment from which the plants were collected. The Normalized Difference Vegetation Index (NDVI) values range from −1 to +1, and values closer to +1 indicate denser and healthier vegetation. For each season, the NDVI values of the collection points were calculated. The results displayed in Figure 11 indicate higher NDVI values during the rainy season regardless of the location of collection. This observation was, however, expected given that vegetation benefits from rainfall. A higher artemisinin content (trend) in the rainy season was associated with the stronger antimalaria activity for *A. annua*. For *A. afra*, its activity varied depending on the season and location of collection. The East collection point exhibited the lowest artemisinin content and had the least suitable vegetation environment, regardless of season.

## 3. Discussion

Plants’ metabolic composition is greatly influenced by geographical location and season, thus directly affecting their potential therapeutic applications [18]. In Cameroon, like in other African countries, *A. annua* and *A. afra* are cultivated throughout several regions and formulated as herbal tea for the management of malaria. However, this is without any pharmacological quality control. This problem is further aggravated by the close morphological similarity between the two *Artemisia* species, making herbal tea’s authenticity problematic. This work focuses its attention on the phytochemical diversity of both species, aiming to discern potential variations in their phytochemical profiles attributable to geographic factors and seasons and correlated with potential pharmacological quality. Several analytical techniques, such as TLC, HPLC, and GC, were employed for the analysis of their metabolic contents, and by using both geographical and multispectral remote sensing data, correlations were made between the observed metabolic fingerprints and anti-plasmodial activity. The results from this study revealed notable variations in the phytochemical compositions of *A. annua* and *A. afra* among different geographical locations and seasons in Cameroon. These variations encompassed differences in the abundance of artemisinin and other secondary metabolites.

*A. afra* and *A. annua* crude extracts are composed of complex mixtures of secondary metabolites, with varying biological activities. The distinctions in the chemical patterns of the semi-volatile and polyphenol constituents observed using TLC, HPLC, and GC analyses between both species could serve as important tools for the correct identification of these medicinal products. For instance, the presence of rutin, astragalin, and luteolin in *A. afra* and their absence in *A. annua*, including the presence of the specific TLC bands in *A. afra*, could be used as a basis for the development of specific monographs for this plant species. Additionally, the TLC fingerprints show the presence of artemisinin specifically in *A. annua*. Intra-species variation in terms of the occurrence and relative composition of plant metabolites, especially in the semi-volatile components, result from the influence of different environmental factors, which vary between different regions and seasons.

Artemisinin is a is a well-known antimalaria compound with a sesquiterpene backbone originally isolated from *A. annua* [14]. Also, traces of artemisinin have been found in some cultivars of *A. afra,* while other cultivars show no detectable content [9,15]. In this study, traces of artemisinin were detected in some *A. afra* samples, while others were devoid of this sesquiterpene lactone. The high artemisinin content in the *A. annua* samples obtained during the rainy season indicates the seasonal influence on the metabolic pathways involved in the synthesis of artemisinin. The rainy season is the growing season during which most of the plant’s secondary metabolites are produced, resulting in alleviated concentrations. Regardless of the season, the artemisinin contents were high in the samples obtained from the Adamawa region, highlighting the effect of both season and geographical location on artemisinin content.

Three different sets of sample preparation were performed for the analysis of the total phenolic contents (TPCs): heating and reflux (using the protocol of the European Pharmacopoeia), methanol crude extracts, and infusion (the traditional preparation). The TPC was greatly influenced by the sample preparation. The highest TPC was retrieved in the methanol extract, followed by the method from the pharmacopoeia, while infusion showed the lowest content. This may be explained by the fact that methanol is a good extraction solvent for most of the polyphenolic components. The higher TPC in the extracts obtained through heating and reflux of the plant powder compared to that in the infusions may have been due to the longer contact time involved in using the former. From this finding, the TPCs, including the contents of CA, tend to be higher in dry season samples of *A. afra* (with the exception of the samples from the Center region), again indicating the seasonal influence.

The acetone and methanol crude extracts prepared from the plant samples obtained both in the rainy and dry seasons from the five geographical regions were tested against a chloroquine-sensitive 3D7 *P. falciparum* strain. Three independent experiments were conducted, and the mean IC_50_ values were calculated and expressed in μg extract/mL. Based on the World Health Organization’s guidelines [8], anti-plasmodial activity levels are classified as follows: highly active (IC_50_ ≤ 5 μg/mL), good activity (IC_50_ between 5 μg/mL and 15 μg/mL), moderate activity (IC_50_ between 15 μg/mL and 30 μg/mL), weak activity (IC_50_ between 30 μg/mL and 50 μg/mL), and a lack of activity at IC_50_ ≥ 50 μg/mL. The anti-plasmodial assays demonstrated varying degrees of efficacy in inhibiting *Plasmodium* parasites, with potential correlations with the distinct phytochemical profiles observed. The acetone extracts were more active than the methanol extracts. This may have been because of the higher artemisinin contents in the acetone extracts than that in the methanol extracts. Certain geographical locations exhibited higher anti-plasmodial activity, indicating a correlation between the phytochemical composition and anti-plasmodial properties. Both samples were active, but the *A. annua* extracts showed a significantly higher anti-plasmodial activity compared to that of *A. afra*. This activity was artemisinin-dependent and stronger in the rainy season samples, being associated with a high artemisinin content. Additionally, the samples from the Adamawa region showed the highest artemisinin contents and were, together with the *A. annua* samples from the South and the East, the most active ones. These three types of samples showed comparable IC_50_ values, though the latter yielded a lower artemisinin content. This indicates that the other components present in the extract also affect its anti-plasmodial activities. Again, this indicates the importance of geographical location and season when cultivating an *Artemisia* sp. for use as a preventive or curative therapeutical agent against malaria. However, WHO has cautioned against the use of non-pharmaceutical sources of artemisinin because of the risk of delivering sub-therapeutic doses, which could exacerbate resistance to ACTs [19], emphasizing the importance of species-specific differences. Despite the lack of relevant contents of artemisinin, *A. afra* showed promising activity against the *Plasmodium* parasites, suggesting its use without the above-mentioned threat to ACTs in emptying malaria reservoirs observed by WHO. The activity of *A. afra* was not affected by season or geographical location despite the relatively higher CA contents during the rainy season.

According to the findings of Ferreira et al. [20], the best growing conditions for Artemisia are a humid and subtropical monsoon climate with average temperatures of 17.6–28.4 °C. Despite the different collecting sites being characterized by a similar climate, the retrieved NDVI values indicate significant variations in the environmental conditions between them. As analyzed using both geographical and multispectral remote sensing data, the rainy season presented healthier vegetation, which favored the artemisinin contents responsible for the greater activity in *A. annua* from this time point than that harvested in the dry season.

## 4. Materials and Methods

### 4.1. Chemicals and Equipment

The reference substances used included artemisinin (Gibco, Fisher Scientific, Merelbeke, Belgium), 1, 8- cineole, α-thujone, β-thujone, rutin, chlorogenic acid, luteolin, astragalin, apigenin, caffeic acid, kaempferol, scopoletin, iso-chlorogenic acid, vitexin, fraxin, caftaric acid, 7-hydroxy coumaric acid, esculetin, quercetin, camphor, coumaric acid, and camphene (Merck, Darmstadt, Germany). The solvents applied—acetonitrile, methanol, formic acid, acetone, ethyl acetate, hexane, ethanol, and chloroform (Sigma-Aldrich, Overijse, Belgium)—were all HPLC-grade. Sulfuric acid, polyethylene glycol (PEG), vanillin powder, and diphenyl-borate diamino-ethanol (DPBAE) were obtained from Merck, Darmstadt, Germany.

### 4.2. Description and Collection of the Plant Material

Morphologically, both plants are similar in appearance, growing up to a height of at least 2 m. *A. afra* is a woody perennial herb with finely divided leaves that are oval and have an aromatic smell, are alternately arranged, and are silver-grey in the adaxial regions and light green in the axial regions [5]. *A. annua* is a large, annual, highly aromatic herb with a single stem covered with fine grey-green hairs and alternating aromatic leaves which are deeply dissected. The leaves and twigs of both plants were collected at the flowering stage from five regions (in both the rainy and dry seasons) in Cameroon: Ngaoundere in the Adamawa region (coordinates: 7.328 N, 13.585 E), Bertoua in the East region (coordinates: 4.579 N, 13.677 E), Sangmalema in the South region (coordinates: 2.937 N, 11.986 E), Bafia in the Center region (coordinates: 4.755 N, 11.224 E), and Dschang in the West region (coordinates: 5.446 N, 10.047 E). Voucher specimens were deposited at the Limbe botanical gardens, Cameroon. The samples were authenticated by a botanist and voucher numbers assigned (SCA 7137 and SCA 6340 for *A. annua* and *A. afra*, respectively).

### 4.3. Preparation of the Crude Extracts

Two groups of plant materials were experimented with: rainy and dry season samples comprising acetone and methanolic crude extracts of four groups of 40 plant samples (20 *A. afra* and 20 *A. annua* samples obtained from five regions). Extracts were prepared following the protocol described by Mbah et al. [21]. Briefly, each plant sample was oven-dried at 30 °C and ground into a fine powder, and each powder was divided into two equal masses and macerated at room temperature for 3 days using acetone or methanol. The macerated mixture was filtered through a funnel stuffed with Whatman filter paper No.1, and the filtrate was concentrated under a vacuum. Each crude extract was then dried and weighed and the yield calculated **(**Supplementary Table S2). All extracts were stored at 4 °C for subsequent use.

### 4.4. Thin-Layer Chromatography (TLC) Analysis

Analytical Thin-Layer Chromatography (TLC) of the extracts was performed on precoated Si gel 60 F_254_ (Merck, Hohenbrunn, Germany) plates to determine the chemical patterns of the compounds in the extracts. A solvent system comprising n-hexane/ethyl acetate (70:30) was used to separate the semi-volatile compounds in the acetone extracts. After development, the TLC plates were sprayed with sulfuric vanillin and heated for 10 min at 100 °C, and the separated components were visualized in daylight. Separation of the polyphenol fraction in the methanol extracts was achieved using a solvent system comprising ethyl acetate, methanol, water, and formic acid (50:4:4:2.5). After development, the results were visualized through DPBAE/PEG spraying (prepared by dissolving 10 g and 50 g of DPBAE and PEG, respectively, in 1 L of methanol) and 366 nm illumination (UV light). The retention factor (RF) of the separated components was calculated and compared with the reference compounds.

### 4.5. HPLC Analysis

High-Performance Liquid Chromatography (HPLC) analyses of the polyphenols were performed on an Agilent 1260 Infinity II system using a protocol developed in the laboratory (Pharmacognosy, CIRM, University of Liège, Belgium). Briefly, the extracts were reconstituted at a concentration of 40 mg/10 mL of methanol, sonicated for 2 × 15 min, and filtered through a 0.45 μm HPLC filter. A Luna PFP column (250 × 4.6 mm, 5 μm) was used, and the mobile phase consisted of a mixture of acetonitrile (A) and water/0.1% formic acid (B) in gradient conditions (A/B: 15/85, 18/82, 23/77, 30/70, 40/60, 50/50, 80/20, 100/0, 15/85, and 15/85 *v*/*v*). A volume of 10 μL was injected at a flow rate of 1 mL/min, and a run time of 87 min was set. A reference calibration curve consisting of chlorogenic acid (0.1, 0.05, 0.025, and 0.0125 mg/mL in methanol) was made. A Diode Array Detector (DAD) was used and set at 330 nm. The results were expressed as chlorogenic acid equivalents.

### 4.6. Analysis of Semi-Volatile Components Using GC

A Gas Chromatography (GC) analysis of the semi-volatile components was performed on an Agilent Technologies Intuvo-9000 GC System using a method developed in the laboratory. Briefly, 100 mg of the samples was weighed and injected using the head-space method. The column used was a DB-WAX-UI (60 m × 250 μm × 0.25 μm). The analytical conditions included an oven temperature programmed from 45 °C to 250 °C at a rate of 2.615 °C/min, and the vial and transfer line temperatures were set at 130 °C and 150 °C, respectively. The chromatogram ran for 120 min, and detection was performed using a Flame Ionization Detector (FID) with a detection temperature of 280 °C. Identification of the different constituents was based on a comparison of the retention times with those of the standard samples.

### 4.7. Quantification of Artemisinin Using the HPLC-UV Method and Derivatization

**Figure 12:**
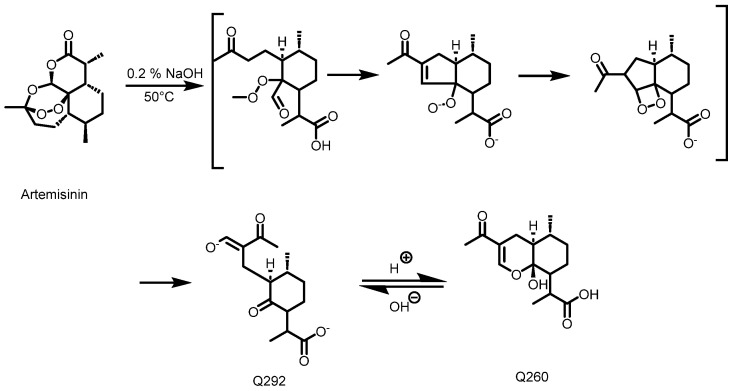
Schematic diagram of artemisinin’s conversion into Q260 [23].

Detection and quantification of the artemisinin in the acetone and methanol crude extracts were performed using an HPLC-UV method after a pre-derivatization step, as described by Diawara et al. [22] and Zeyuan et al. [23]. Before the pre-derivatization step, 50 mg of crude extract was dissolved in 5 mL of ethanol and submitted to 2 × 15 min of ultrasonication. For all of the samples including the standards, 1 mL of solution was transferred into a 10 mL volumetric flask, followed by the addition of 4 mL solution of 0.2% (m/v) NaOH, leading to the formation of Q292 (Figure 12) after 30 min in a water bath at 50 °C. The mixture was cooled for 10 min, 1 mL of ethanol was added, and the flask was topped up to a mark (25 mL) with 0.2 N acetic acid, promoting the conversion of Q292 into Q260. Figure 2 illustrates the derivatization reaction which produces Q260, as detected at 260 nm following the HPLC analysis. All of the samples were filtered using a 0.45 μm HPLC filter, and chromatographic separation was performed in reverse phase mode. A mobile phase consisting of a mixture of methanol and phosphate buffer (5.0 mM; pH: 6.3) (45/55 *v*/*v*) was used. The Luna PFP column (250 × 4.6 mm, 5 μm) was maintained at 35 °C. An injection volume of 20 µL and a flow rate of 1 mL/min with a run time of 25 min were set as the chromatographic conditions. A calibration curve consisting of 10, 20, 40, and 60 mg/mL of artemisinin was used to estimate the quantity of artemisinin in each extract.

### 4.8. Quantification of Polyphenols

The total polyphenol contents (TPCs) in the plant powder, infusions, and plant extracts (methanol extracts) were determined spectrophotometrically using the method described in the European Pharmacopeia [15]. Briefly, 500 mg of plant powder was weighed in a round-bottomed flask, and 100 mL of water R was added. The flask was heated and refluxed for 30 min and then cooled under running water and filtered into a 100 mL volumetric flask. The infusions were prepared by adding 5 g of plant powder into 1 L of water and left to extract for 15 min. Stock solutions of the methanol crude extracts were prepared by dissolving 20 mg of crude extract in 10 mL of methanol and ultrasonicating them for 15 min.

For quantification, 2 mL of each solution was transferred into a 25 mL volumetric flask, and 1 mL of phosphomolybdotungstic reagent and 10 mL of water R were added and adjusted to 25 mL with a solution of sodium carbonate R. After 30 min in the dark, the absorbance was measured at 760 nm using a U-2900 spectrophotometer. Pyrogallol was used as a reference. A stock solution was prepared by dissolving 5.23 mg of pyrogallol in 100 mL of methanol and quantified as described above. The results were expressed as pyrogallol equivalents.

### 4.9. Anti-Plasmodial Activity

A chloroquine-sensitive 3D7 *P. falciparum* strain was obtained from the Malaria Research and Reference Reagent Resource Center, MR4, and maintained in vitro as described by Trager and Jensen [24]. The parasites were maintained at 3% hematocrit (human type A- or O-positive red blood cells) in a Complete Culture Medium (CCM) prepared by supplementing RPMI 1640 (Gibco, Fisher Scientific, Merelbeke, Belgium) medium containing NaHCO3 (32 mM), HEPES (25 mM), and L-glutamine with 10% heat-inactivated human plasma, 1.76 g/L of glucose (Sigma-Aldrich, Overijse, Belgium), 44 mg/mL of hypoxanthine (Sigma-Aldrich, Overijse, Belgium), and 100 mg/L of gentamycin (Gibco, Fisher Scientific, Merelbeke, Belgium). All of the cultures were placed in a humidified incubator at 37 °C with a standard gas mixture of 5% O_2_, 5% CO_2_, and 90% N_2_. Parasitemia was verified through microscopy using Giemsa-stained thin films. A stock solution of the crude extracts was prepared by dissolving 5 mg of extract in 1 mL DMSO and diluting it with culture media. The anti-plasmodial test was performed in 96-well microtiter plates at an initial sample concentration of 50 μg/mL which was serially diluted to eight concentrations (50 to 0.391 μg/mL). Each sample was tested in duplicate wells with the parasites at a final volume of 250 μL, making the final DMSO concentration <1%. Wells with parasites only served as the positive control, while the negative control consisted of wells with the culture medium only. A 1 μg/mL stock solution of artemisinin (Gibco, Fisher Scientific, Merelbeke, Belgium) was prepared and serially diluted as above; this was used as the standard. After 48 h of incubation, parasite growth was estimated through determination of the plasmodial lactate dehydrogenase activity, as described by Kenmogne et al. [25], and the IC_50_ values were calculated through linear regression. All tests were repeated at least twice.

### 4.10. Multispectral Satellite Imagery Analysis of the Health Status of Vegetation at the Collection Sites

The health status of the vegetation was monitored using Earth observation. The pair of Sentinel-2 satellites from the European Copernicus Programme (ECP) provides free images of the Earth’s entire surface at a spatial resolution from 10 to 60 m. Each Sentinel-2 satellite carries a Multispectral Instrument (MSI) that captures images in 13 spectra bands, covering the visible, near-infrared, and shortwave infrared regions (443–2323 nm). Multispectral remote sensing data are widely used for land and ocean monitoring using spectral indices. To assess the vegetation’s health and density, the Normalized Difference Vegetation Index (NDVI) was derived from the reflectance, i.e., the solar light reflected by the Earth’s surface, in the red and near-infrared (NIR) bands using the following formula:

NDVI = (NIR − Red)/(NIR + Red).

The NDVI values range from −1 to +1. Vegetation absorbs most visible light for photosynthesis (implying a low reflectance in red) and reflects a significant amount of near-infrared light (a high reflectance in NIR), leading to a positive NDVI. Denser and healthier vegetation presents an NDVI closer to +1. For each season, the NDVI values at the collection points were calculated using cloud-free Sentinel-2 images acquired on the dates closest to the collecting dates (Supplementary Table S1). Given the spatial resolution of Sentinel-2 images in the visible wavelengths (10 m), the NDVI was not retrieved at the plant level but at the level of the direct environment in which the plants grew.

## 5. Conclusions

The results show both intra-species and inter-species variations in the phytochemical constituents which fluctuate considerably with season and geographical region. The artemisinin content was found to be high in the rainy season for *Artemisia annua*, while the polyphenol contents and chlorogenic acid and its derivatives were high in the dry season for *Artemisia afra*. The observed variations may have relevant implications for the anti-plasmodial potential of these plants, with specific geographic factors influencing their chemical composition, and highlight the importance of quality control in herbal medicine. The findings suggest that the selection of a suitable *Artemisia* sample for use as a potential antimalarial treatment must take into consideration its geographical origin, the period of collection, season, and remote sensing data. By understanding the environmental factors that influence the phytochemical composition of *Artemisia* samples using remote sensing data, this study may provide an insight into how natural products can be more effectively used as a preventative or curative measure against malaria. Further research is warranted to elucidate the major compounds responsible for the observed anti-plasmodial activities in *A. afra* and the effect of post-harvest handling on its secondary metabolic contents.

## Data Availability

Data are contained within the article and Supplementary Materials.

## Supplementary Materials

The following supporting information can be downloaded at https://www.mdpi.com/article/10.3390/molecules30030596/s1. Figure S1: Field pictures of (a) A. annua and (b) A. afra. Figure S2: TLC fingerprint: (a) Acetone extract of A. afra and A. annua obtained in the rainy season revealing variations in the terpene chemical composition. (b) Acetone extract of A. afra obtained from different regions showing similar chemical parttern of terpene composition, (c) Variation in polyphenol chemical pattern between both species (methanol extract of A. afra and A. annua) obtained in the rainy season, (d) Methanol extract of A. afra and A. annua obtained in the dry season revealing polyphenol chemical pattern. X = scopoletin, Y = artemisinin and Z = apigenin. Figure S3: Contents of ART in acetone and methanol extracts of samples from the Center and Adamawa regions. Figure S4: TPCs in the different methods of extract preparation (heating and reflux method proposed by European pharmacopoeia, tea infusion extract and methanol extracts. Table S1: List of the collected sites indicating the collection dates of the samples for both seasons and the dates of the closest sateniel-2-images used for inferring the NDVI. Table S2: Yield of plant extracts.
