# Supplementary material for: Exploring the Phytochemical Diversity and Anti-Plasmodial Potential of Artemisia annua and Artemisia afra from Different Geographical Locations in Cameroon
Source: Molecules. 2025 Jan 28;30(3):596. doi: 10.3390/molecules30030596 (PMC11819670; doi:10.3390/molecules30030596)
Supplement: Supplementary file 1 [file molecules-30-00596-s001.zip › molecules-3396406-supplementary.pdf]

SUPPLEMENTARY MATERIAL

A B

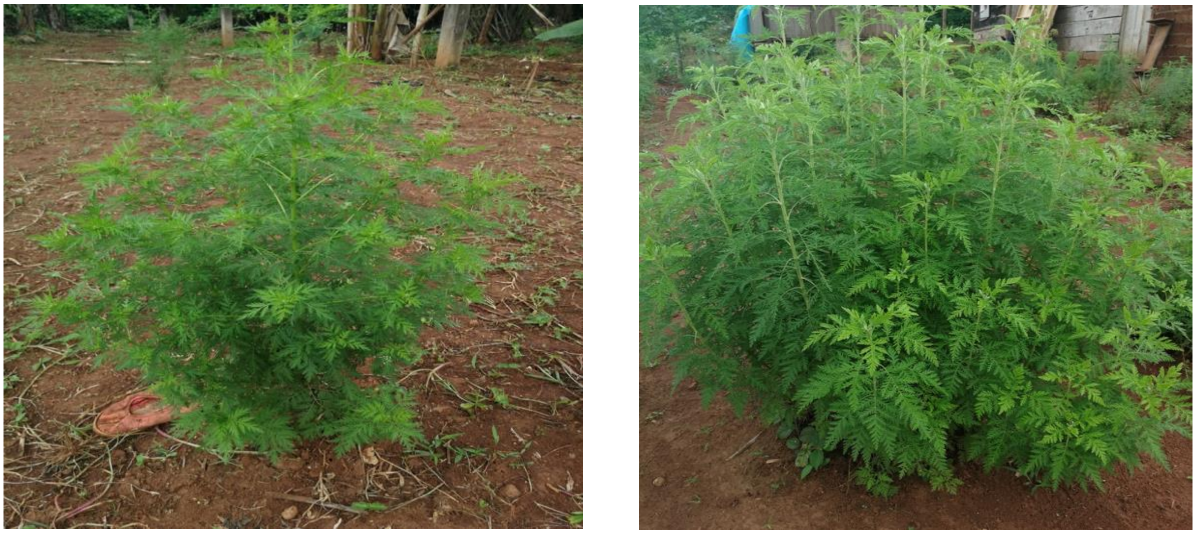

Supplementary Figure S1: Field pictures of (a) *A. annua* and (b) *A. afra*

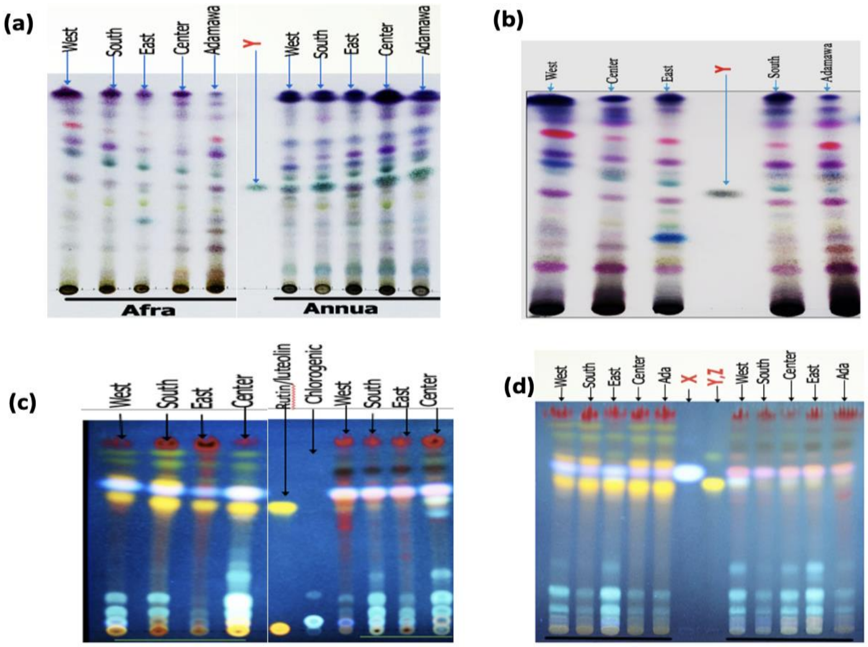

Supplementary Figure S2: TLC fingerprint: **(a)** Acetone extract of *A. afra* and *A. annua* obtained in the rainy season revealing variations in the terpene chemical composition. **(b)** Acetone extract of *A. afra* obtained from different regions showing similar chemical parttern of terpene composition, **(c)** Variation in polyphenol chemical pattern between both species (methanol extract of *A. afra* and *A. annua*) obtained in the rainy season , **(d)** Methanol extract of *A. afra* and *A. annua* obtained in the dry season revealing polyphenol chemical pattern. X= scopoletin, Y= artemisinin and Z= apigenin.

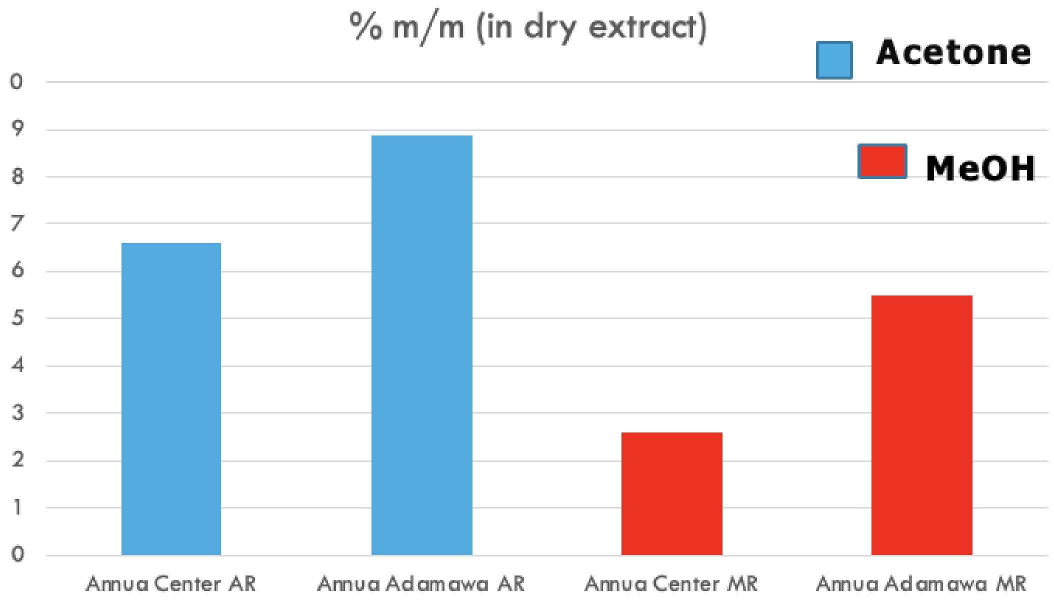

Supplementaruy Figure S3: Conetents of ART in acetone and methanol extracts of samples from the Center and Adamawa regions.

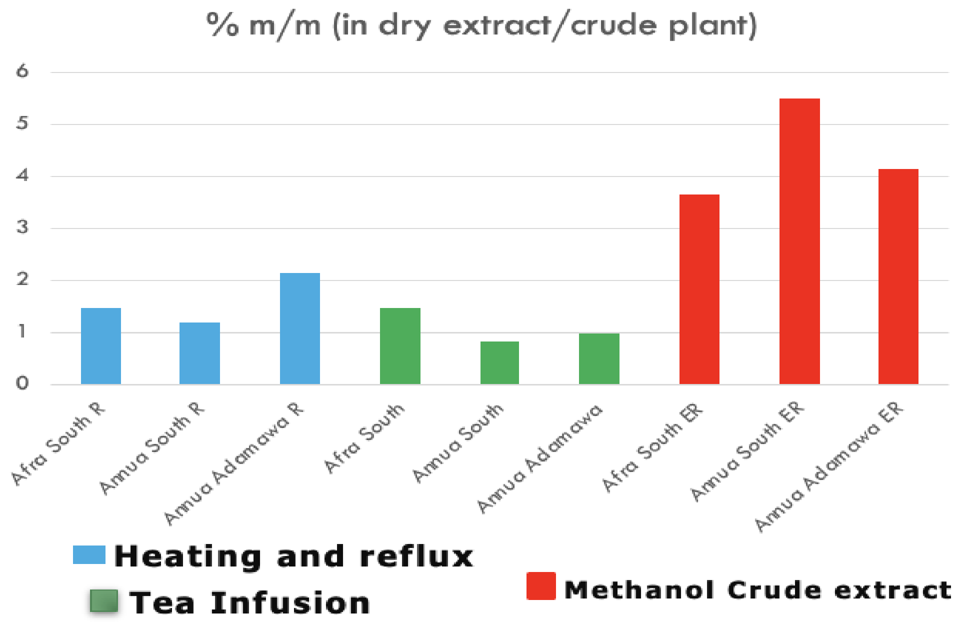

Supplementary Figure S4: TPCs in the different methods of extract preparation (heating and reflux method proposed by European pharmacopoeia, tea infusion extract and methanol extracts

Supplementary Table S1: List of the collected sites indicating the collection dates of the samples for both seasons and the dates of the closest sateniel-2-images used for inferring the NDVI.

Regions	Town	Date of collection		Date of Sentinel-2 images	
		Rainy season	Dry season	Rainy season	Dry season
South	Sangmalema	28-07-21	21-04-22	10-09-21	29-03-22
West	Dschang	07-07-21	13-04-22	25-06-21	06-04-22
East	Bertoua	19-07-21	07-04-22	06-08-21	19-03-22
Center	Bafia	11-07-21	15-04-22	20-07-21	01-05-22
Adamawa	Ngaoundere	26-07-21	17-04-22	22-07-21	08-04-22

Supplementary Table S2: Yield of plant extracts

	Artemisia afra				*Artemisia annua*			
**Region**	**YA/g**	**%YA**	**YM/g**	**%YM**	**YA/g**	**%YA**	**YM/g**	**%YM**
West	4	6	7	9	7	12	5	10
Center	11	10	8	11	10	10	9	12
East	11	13	8	9	5	6	8	10
South	10	8	9	13	11	11	10	7
Adamawa	9	10	10	12	7	10	11	15

YA= Yield of acetone extract, YM= Yield of methanol extract.
